# Supplementary material for: Caregiving motivations and experiences among family caregivers of patients living with advanced breast cancer in Ghana
Source: PLoS One. 2020 Mar 12;15(3):e0229683. doi: 10.1371/journal.pone.0229683 (PMC7067415; doi:10.1371/journal.pone.0229683)
Supplement: S1 File — (DOCX) [file pone.0229683.s001.docx]

**Title of study: Caregiving motivations and experiences among family caregivers of patients living with advanced breast cancer in Ghana.**

**Interview process**

Thank you for accepting to share your caregiving motivations and experiences of living with advanced breast cancer women. Before we begin our discussion, I want to guarantee you that your interview responses will be kept confidential. Together with that of other participants, your responses will not be associated with your name or any recognizable information that can be used to trace you.

You may refuse to respond to any questions you don’t want to answer and you may end the interview at any time. You may also decide to quit this study altogether at any time without any consequences. We will endeavor to record this interview to ensure that your responses are accurately listened to. All your responses will be written down verbatim to help support our findings based on the interpretation of your comments.

The recordings and the transcripts will be shared with a person who is good in Twi and English language to assess your responses which we have written down. This will ensure that we have written your responses accurately. Also, your written responses will be shared with the research team for analysis and validation. Again, you are reassured that your name will not be linked to the recording or the transcript. A study code or number will be allocated to you. Before sharing with other individuals who may have access to the study data, your name or any recognizable information that comes on the transcript will be deleted.

As part of the study or during publication of any aspects of the study, some of your expressions may be quoted. Therefore, we will use a false name in place of your allocated number when quoting you. All your recordings and transcripts will be safely kept with a password and in a cabinet under lock and key for 5 years. Any of your documents bearing your recognizable information will be kept separately. Therefore, no one will be able to trace the documents to you.

At the end of the storage period, all the documents will be destroyed. You will have to sign or thumbprint a consent form which I will witness, showing your readiness to get involved in the study.

Do I have your permission to start the interview?

[If no, thank the participant for time and end the session] [If yes, continue with the interview].

Thank you. Before I begin the interview, pleases sign the consent form.

Thank you for consenting to participate in this study. Before I begin the recording, I will ask some general questions about you.

# Interview Guide

**Section A: Background Information Form**

1. Age…………………………………
2. Gender: Female [ ]; Male [ ]
3. Place of residence………………………………………………….
4. Nationality ……………………………………………………........
5. Marital status: Married [ ]; Divorced [ ]; Never married [ ]; Separated [ ]
6. Number of children…………………………………………………
7. Occupation …………………………………………………………
8. Level of education …………………………………………………
9. Language(s) spoken …………………………………………………
10. Religion …………………………………………………………….
11. Relationship to care recipient……………………………………..
12. Length of caregiving ………………………………………………
13. How long have your relative been diagnosed with breast cancer? .........................
14. Stage of the breast cancer…………………………………………………..

**Section B: Guiding Questions**

Main question:

Please share with me your caregiving motivations and experiences as a family caregiver of a patient living with advanced breast cancer.

Probe

1. Can you please describe what motivated you to take on the caregiving role?

- Your relation
- Factors

1. Can you please tell me more about how you care for, manage and monitor the patient at home?

- Common complaints, type and features
- Symptoms? Type? Nature of symptoms
- Observation and Management strategies

1. Please, share with me about your day-to-day caregiving experiences at home for the patient.

- Feeding?
- Bathing?
- Grooming?

1. What other needs of the patient do you meet?

- Visiting the hospital
- Finance
- Social
- Emotional
- Spiritual
- Strategies used

1. Is there anything else you would like to share with me?

Thank you.
