# Supplementary material for: Caregiving motivations and experiences among family caregivers of patients living with advanced breast cancer in Ghana
Source: PLoS One. 2020 Mar 12;15(3):e0229683. doi: 10.1371/journal.pone.0229683 (PMC7067415; doi:10.1371/journal.pone.0229683)
Supplement: S2 File — (DOCX) [file pone.0229683.s002.docx]

| **THEMES** | **SUB -THEMES** | **EXAMPLE QUOTES** |
| --- | --- | --- |
| **Motivation for assuming the**  **caregiving role** | Caregiving as a family and socio-cultural obligation  Caregiving as a sign of reciprocity | *“She is my mother, my family… it is actually my socio-cultural responsibility to take care of her… That is the reason I am the one taking care of her” (Participant 14, Son).*  *“As our culture demands, I have to take care of her. It is just my duty as her (patient) family member” (Participant 7, Sister).*  *“Hmmm..! As the eldest daughter, it is my cultural duty to take care of her. I have no other option than to take care of her” (Participant 12, daughter).*  *“I am a woman and her mother and it is my cultural duty to take care of her” (Participant 11, Mother).*  *“Oh! She is my wife… if I don’t take care for her, who will? It is just my social obligation as a husband to take care of her” (Participant 10, husband).*  *“When she was diagnosed of the disease (breast cancer) she had no one to take care of her, hence, as a brother, it is culturally expected that I take care of her. There are no women around, hence I have to do it” (Participant 3, brother).*  *“All my sisters are currently not in Ghana. As the only male available, I have to take care of my mother because is socially expected of me “(Participant 13, son).*  *“It is unusual for someone who is not your family member to take care of you when you are sick, but she (patient) did it passionately when I was admitted at the hospital. So now that she is sick, it is my turn to repay her for what she did for me” (Participant 9, Friend).*  *“She used to support me by lending me money when she was working. So now that she is sick, I have to also support her till she recovers from this illness. I am just paying her back for all the support she has been giving me” (Participant 15, Friend).*  ” *she is the bone of my bone because she made the same sacrifice for me when I was sick. Likewise, in this situation, I have to sacrifice for her*”. (participant 4, husband)  “*She gave birth to me and supported me during my difficult times. hence, I have to leave whatever I am doing and take care of her. It is just a kind of repayment of all the good she has done for me” (Participant 2, Son)* |
| **Meeting self-care and**  **psychosocial needs of patients** | Assisting with activities of daily living  Spiritual support  Emotional support  Financial support | *“I fetch and boil water for her every day. I also groom her every day because she becomes very weak whenever she goes for therapy (chemotherapy)” (Participant 14, Son).*  *“She cannot wash her dirty cloths because her hand is always swollen and heavy (lymphedema). So, I am the one who does all her laundry” (Participant 3, brother).*  *“Her blood level has gone low, now she is weak and she cannot do anything for herself. She cannot bath, I bath her…I even have to walk her to the toilet sometimes” (Participant 5, Sister).*  *“She is always in pain because of the breast wound and she cannot wash her cloths. I therefore wash her cloths” (Participant 11, Mother).*  *“I bath her, do all the groceries. She cannot do anything, she just can’t do anything because of her swollen arm (lymphedema)” (Participant 4, husband).*  *“Because of her breast wound and her swollen arm, she is not able to go out, so now it is my duty to buy foodstuffs from the market and prepare food for her in the morning and evening”(Participant 12, daughter).*  *“I pray for her and share healing messages in the Bible with her. This has really helped her to have some inner peace now” (Participant 11, Mother).*  *“Now she (patient) does not cry anymore because I always encourage her that God is on the throne and that He will heal her. I pray and share God’s words with her. These have really increased her faith in God” (Participant 4, Husband).*  *“I always remind the church leaders every month about the Holy Communion. I make sure that they always bring her bread and wine in the house” (Participant 9, Friend).*  *“Sometimes, I arrange pastoral visits for her. The Osofo (Reverend) comes in to pray with her (Participant 13, Brother).*  *“Because of the cancer, sometimes when she cries, I always feel like crying. However, I get the courage and I always console her and tell her that I will always support her through her illness and if she has any problem, she shouldn’t hesitate to tell me. This really helps with her worries”* (Participant 4, husband).  *“She was always thinking about the disease, but I encourage her with cheerful and hopeful messages to relieve her emotional distress” (Participant 6, Sister).*  *“I always make sure that I communicate with her and encourage her to forget about the breast cancer so that she can be happy all the time” (Participant 15, Friend).*  *“Although I worry a lot about her condition every day, I try to be cheerful when I am with her. I get time to listen to her and console her when she is lonely” (Participant 3, Brother).*  *“ I usually encourage her by assuring her that she will be healed from this illness. I also told her that she shouldn’t worry since it has affected only the right breast” ( Participant 13, Brother ).*  *“ I always get the courage to offer words of encouragement to her. Everyday I give her that emotional support so that becomes relaxed (Participant 11,Mother)*  *“At the hospital, all the little money on me has been spent on her treatment because the NHIS covers only the folder and some of the infusions but all the other medications, I have to buy them” (Participant 1, Husband).*  *“The National Health Insurance Scheme (NHIS) does not cover all the cost of the treatment. So, the little I get, I spend it all on my mother’s drugs and living expenses” (Participant 2, Son).*  *“I provide money for everything including transportation to the hospital. Where we live, if we take a taxi to the hospital, they charge GHC 120 (≈23 USD) for our round trip” (Participant 10, Husband).*  *“Aside money for her drugs, I always buy food supplements (Forever Living) to meet her nutritional needs. Although expensive, I manage to buy it for her” (Participant 15, Friend).* |
| **Symptom management and monitoring** | Home-based wound care  Management of breast cancer-related lymphedema | *“We went to an herbalist who gave us a mixture of charcoal and clay to apply on the wound to help with the bleeding. It really helped with the bleeding and helped in removing the sloughs. So I use it for the dressing at home” (Participant 11, Mother).*  *“I use to dress the wound or her sister. What we do is that we put salt in lukewarm water and we go to the herbal shop to buy a herbal ointment to apply on her wound” (*Participant 10, Husband ).  *“I sent her to an herbal center at Asokwa. The herbal doctor usually grinds some green leaves for us to apply on the wound at home and it has really reduced the mal-odor” (Participant 9, Friend).*  *“In the house I dress her wound for her. I use salt water and then apply specially prepared ointment (chloramphenicol and penicillin ointment) the wound”. (Participant 4*)  *“Her arm is always heavy so I usually apply bandages and put the arm in a sling for her. Now the weight has started reducing gradually” (Participant 12, Daughter).*  *“When her arm becomes swollen, I make sure I elevate the affected arm by putting the arm on pillows. This has helped in decreasing the swollen arm” (Participant 4, Husband).*  *“Her arm was always swollen and I sent her to an herbalist who put some herbs in a horn and blew it on the swollen arm” (Participant 10, Husband).*  *“I usually mix charcoal with clay and apply it on her (patient) swollen hand and by the next day, the hand will be better. So, that is what I have been using for the hand” (Participant 11, Mother).*  *“Especially when her arm becomes swollen (lymphedema), I used some herbs and apply it on it. So it was the herbs that I was using” (Participant8, daughter).* |
|  | Drug administration and pain management | *“Per the doctor’s instruction, she was supposed to take 100 mg tramadol 2 times daily and 1 gram paracetamol three times daily. But the pain was still unbearable so I had to give her more than the prescribed medicine. Now, I give her 200mg tramadol to relieve her pain and also help her to sleep” (Participant 12, Daughter).*  *“One tablespoon (10mg) Morphine 3 times daily is what they (doctors) prescribed for her. But, normally, she complains of pain in the breast. The breast is ulcerated so she really feels the pain. So I administer two tablespoons (20mg) to her in the house anytime she complains of pain. I don’t stick to the prescribed dosage because it just couldn’t relieve her pain” (Participant 4, Husband).*  *“Sometimes, she (patient) can ask for the whole bottle of morphine because of the unbearable pain. But I always say no to her request. The doctor says you can experience side effects such as constipation and addiction when you take too much of the morphine” (Participant 8, Daughter).*  *“Due to this, I do not administer the drug daily per the prescription … I administer the drug to her when the pain is too much” (Participant 11, Mother).*  *“Where we were staying, we had no fan. So whenever she cried and complained of pain, I fan her to reduce the pain and further promote her comfort” (Participant 10, Husband).*  *“Sometimes when she complains of pain, I intentionally play her favorite program on TV for her to watch and it really helps to take her mind off the pain” (Participant 5, Sister).* |
|  | Continuous evaluation of symptoms and patient advocacy | *“After the first cycle of chemotherapy, they told us she will vomit and also have diarrhea. But the vomiting was too much for three consecutive days. So, I called the doctor and told him about it and he said it is the side effect of the chemotherapy. But I told the doctor that the vomiting was too much. So, he told us to send her to the nearest hospital” (Participant 6, Sister).*  *“I don’t know whether it was because of the drugs she was taking. She was always complaining “my stomach, my stomach”. When she complains of the stomach pains, she usually feels like vomiting so I called the doctor and told him about what was happening” (Participant 11, Mother).*  *“Following her first cycle of her chemotherapy, her wound seemed reducing in size but I don’t know what happened….., her wound just started getting worse when she took the third cycle.. I reported to the doctor at our review so he could do something about it”* (Participant 5, Sister). |
