# Supplementary material for: Caregiving motivations and experiences among family caregivers of patients living with advanced breast cancer in Ghana
Source: PLoS One. 2020 Mar 12;15(3):e0229683. doi: 10.1371/journal.pone.0229683 (PMC7067415; doi:10.1371/journal.pone.0229683)
Supplement: S1 Table — (DOCX) [file pone.0229683.s003.docx]

**S1 Table: Demographic characteristics of participants**

| **Participant**  **Number** | **Age**  **(years)** | **Marital Status** | **Number of Children** | **Educational**  **Background** | **Religion** | **Occupation** | **Relationship to Patient** | **Length of caregiving** | **Stage of care recipient** |
| --- | --- | --- | --- | --- | --- | --- | --- | --- | --- |
| 1 | 57 | Married | 3 | Ordinary level | Christian | Business Man | husband | 1 ½ years | Stage IV |
| 2 | 45 | Married | 3 | Basic | Christian | Driver | Son | 3 years | Stage IV |
| 3 | 53 | Married | 3 | Basic | Christian | Driver | Brother | 10 months | Stage III |
| 4 | 56 | Married | 4 | Ordinary level | Christian | Pastor | Husband | 2 years | Stage IV |
| 5 | 47 | Single | 2 | None | Christian | Trader | Sister | 1 year | Stage IV |
| 6 | 55 | Married | 3 | Basic | Christian | Trader | Sister | 7 months | Stage IV |
| 7 | 45 | Married | 2 | Ordinary level | Christian | Trader | Sister | 3 months | Stage IV |
| 8 | 32 | Married | 3 | Basic | Christian | Trader | Daughter | 2 years | Stage IV |
| 9 | 48 | Married | 2 | None | Christian | Trader | Friend | I year | Stage IV |
| 10 | 36 | Married | 7 | Tertiary | Moslem | Trader | Husband | 2 years | Stage IV |
| 11 | 73 | Widowed | 4 | Tertiary | Christian | Pensioner | Mother | 3 years | Stage IV |
| 12 | 25 | Single | 0 | Senior high school | Christian | Shop attendant | Daughter | 1 year | Stage IV |
| 13 | 27 | Single | 0 | Senior high school | Christian | Mason | brother | 1 year | Stage III |
| 14 | 28 | Single | 0 | Tertiary | Christian | Student | Son | 3 years | Stage III |
| 15 | 38 | Married | 5 | Basic | Christian | Trader | Friend | 10 months | Stage III |
