# Supplementary material for: Caregiving motivations and experiences among family caregivers of patients living with advanced breast cancer in Ghana
Source: PLoS One. 2020 Mar 12;15(3):e0229683. doi: 10.1371/journal.pone.0229683 (PMC7067415; doi:10.1371/journal.pone.0229683)
Supplement: Attachment — Submitted filename: Response to reviewer final copy.pdf [file pone.0229683.s004.pdf]

Department of Nursing
College of Health Sciences
Kwame Nkrumah University of Science and Technology
Kumasi-Ghana
15th January, 2020

Academic Editor
PLOS ONE

Dear Rosemary Frey

<u>RESPONSE TO REVIEWER COMMENTS- PONE-D-19-26686</u>

We express our appreciation to the editors and reviewers for the constructive critique of our manuscript. Authors agree with reviewers on most of the concerns raised and have addressed comments in the revised manuscript.

In response to comments, we have provided a point by point response to each comment in the table of changes below. Also, where substantive changes, deletions and insertion of key sentences have been made to the revised manuscripts, exact changes and where (pages) effected, have been clearly stated as part of the responses to comments in the table.

In addition to this cover letter, we have provided a clean copy highlighting in green font all changes made to the revised manuscripts as required. Also, a clean revised manuscript without any annotations, highlighting or comments have been added as required.

Thank you

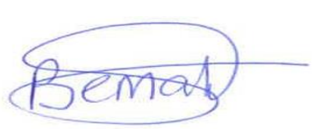

DR (MRS) ADWOA BEMAH BONSU

CORESPONDING AUTHOR

Journal: PLOS ONE

Subject: Responses to Reviewers comments

Table of Changes

Reviewer #	Page Number	Reviewers' Comments	Authors' Responses
Reviewer #1	Data analysis and management.	It is unclear in Data analysis whether the procedure you took is the Colaizzi approach – could you clarify this. Colaizzi is difficult to find on line therefore it needs more clarification and preferably a more recent reference in addition. Also, the reference needs to show it is an edited book but consider using a more up to date reference for your methodology and relate more what you did to the reference you are using.	We thank the reviewer for the important suggestion raised on clarifying the Colaizzi's approach used for data analysis.<br><br>The comment has been addressed and highlighted in green font of the revised manuscript, now page 9-10, line 189-217 to provide more clarification on how the Colaizzi's approach was used.
	Line 55	Word form - Wound	We thank the reviewer for this important comment. We have addressed this comment in page 3, line 55.
	Line 58	article required before treatment – also subject verb agreement – head noun in that sentence is treatment trend (is)	This comment has now been addressed in the revised manuscript in page 4, now line 73
	Line 64	add "as well as"	We thank the reviewer for the observation made.

			We have added as well as in the revised manuscript page 4, now line 84
	Line 72	researches (Count noun?)	This comment has been addressed in page 4, now line 93 of the revised manuscript.
	Line 80<br><br><br><br><br><br><br><br><br>Line 116	Delete article an and add an article in line 116	We thank the reviewer for the observation made. We have deleted the article “an” now in page 5, line 101 of revised manuscript.<br><br><br><br>The article “a” have been added to page 7, now line 137 and the sentence has been reconstructed to make it more meaningful.
	Line 118	delete s off interests	We thank the reviewer for the observation made. We have deleted s off interests in page 7, now line 139 of the revised manuscript.
	Line 151	count vs non-count noun -information	This comment has been addressed in the revised manuscript in page 8, now line 172.
	Line 197	add a reference to confirmability claim	We thank the reviewer for this comment. We have addressed this comment, and added a reference to the confirmability claim in page11, now line 236 of the revised manuscript.
	Line 204	a reference for and more detail of ‘voice-centered data analysis	We thank the reviewer for this comment. This

			comment has been addressed and a reference and a more meaningful statement of data analysis has been added in page 11, line 242-244 of the revised manuscript.
	Table S1	Table S1? Where is it?	We thank the reviewer for this comment. Table S1 is in the additional file that was submitted with the manuscript and has been highlighted in page 12, now line 263 of the revised manuscript
	Line 336	replace these for this	We thank the reviewer for the important observation. We have addressed this in page 17, now line 364 of the revised manuscript.
	Line 345	Cited that?	This has been well elaborated in page 17, now line 371-374 of the revised manuscript.
	Line 443	delete an ‘only’	We thank the reviewer for pointing this out. We agree with the comment. Therefore we have addressed in page 20, now line 460-461 of the revised manuscript.
	Line 475 – 78	Not sure what you are saying here. Sense?	We thank the reviewer for this comment. We have reconstructed the statement into a more meaningful statement in page 21, now line 489 -491 of the revised manuscript.

	Discussion	Discussion - could do with being a bit sharper and to the point.	We appreciate the reviewer's comments. As suggested by the reviewer, we have been sharper in the 'discussion' section in page 22-24, now line 504-562 of the revised manuscript.
	Line 526	I'm not sure the contrast with the Iranian participants adds anything because we aren't told why this is different	We thank the reviewer for this suggestion. the Iranian example has been removed and the statement has been amended as suggested in Page 23, now line 543-546 .
	Line 542	incomplete sentence and is it an irony	We thank the reviewer for the important input. We have reconstructed the sentence into a complete statement to make it more meaningful in page 27, line 554-557
	Line 560	Which home-based model are you referring to? Have you explained it elsewhere in the paper?	We thank the reviewer for the important input. The Home based model have been introduced and well elaborated in the 'introduction section (page 3-4, now line 58-77)'. This has given a context to the home-based care model presented in the "conclusion section" page 25, now line 573 of the revised manuscript .
Reviewer #2		This article makes an important contribution to family and socio-cultural factors in breast cancer caregiving in	We thank the reviewer for this valuable feedback. As suggested by the reviewer, we have accordingly

		Ghana. The paper still needs a bit more proofreading, but is otherwise satisfactory in all major respects and I would recommend publication.	proofread the revised manuscript.
